# Supplementary material for: The impact of intransitivity on the Elo rating system
Source: PLoS One. 2025 Dec 26;20(12):e0338261. doi: 10.1371/journal.pone.0338261 (PMC12742789; doi:10.1371/journal.pone.0338261)
Supplement: Attachment — Submitted filename: Elo_PLOS_One__Response_to_reviewers.pdf [file pone.0338261.s003.pdf]

Response to reviewers

July 2025

1 Response to reviewers

We thank the reviewers for their insightful comments. Their comments were helpful in ensuring that our paper was articulate and readable.

2 Reviewer #1

Thank you for your submission! I have carefully read your article, which focuses on the performance of the Elo rating system when its underlying modeling assumptions are not met, particularly from the perspective of relaxing the transitivity assumption, demonstrating uniqueness.

The Elo rating system is a commonly used statistical method for analyzing paired comparison data. This paper proves that after relaxing the transitivity assumption, the Elo rating system exhibits undesirable characteristics where estimated ratings depend on opponents, a finding that deepens the understanding of its limitations. Current research on the Elo rating system is mostly based on conventional assumptions, whereas this paper breaks new ground by exploring more complex and realistic scenarios, offering practical guidance.
Response: Thank you for acknowledging the originality of our research.

1. Although the introduced statistical measures were tested on simulated data, the paper lacks integration with real-world applications, missing case studies from games or competitions to further validate their effectiveness and practicality. More in-depth and actionable recommendations grounded in real-world contexts should be provided.

Response: We have not been able to find real-world data sets rich enough to demonstrate the phenomena described in our paper. This would involve datasets, that even if the majority of data points were discarded would still achieve convergence. We have gotten around this problem by simulating our data. These simulations give us ground truth (which real data lacks) as well as control over parameters of the systems.

2. The impact of intransitivity on the Elo rating system lacks coverage and consideration of variable factors. Due to the variability of external conditions

and influencing factors, the paper fails to present a comprehensive and authoritative perspective, leading to a partial discussion of the Elo rating system's performance. This may overestimate its actual impact.

Response: Elo, and ratings in general, are complex and there is a large literature dealing with the various factors that impact them. This paper deliberately focusses on the transitivity component which is under-researched at present. However, we have noted your suggestions and have included some additional ideas in the future directions of the work.

3 Reviewer #2: Review Comments to the Author

The manuscript presents a timely and important investigation into how the Elo rating system behaves under intransitive conditions, an area of growing relevance across various domains including machine learning and performance evaluation.
Response: Thank you.

The theoretical contributions are sound, well-motivated, and clearly articulated. The use of combinatorial Hodge theory to model and measure intransitivity is both novel and appropriate.

The paper is generally intelligible and written in standard academic English, but there are several grammatical and stylistic issues that should be addressed to improve clarity and polish.

Notable language issues include subject-verb disagreement (e.g., "the Elo rating system exhibit" instead of "exhibits"), awkward phrasing, and occasional redundancy (e.g., "this point, this point represents…" in the abstract).
Response: Thank you for your attention. We have read through the paper and have eliminated grammatical mistakes and redundancies

Certain sections, particularly in the introduction and background, could be more concise. Repetition of ideas sometimes disrupts the flow and can be edited for clarity.
Response: Certain repetitive sections of the paper have been cut to improve clarity.

Minor formatting inconsistencies are present, likely due to LaTeX typesetting or PDF conversion. These include irregular footnotes, inconsistent citation styles, and occasional spacing issues.
Response: The footnotes have been standardised. likewise, the citations and spacing have been standardised.

Some definitions and theoretical terms (such as "Elotope") would benefit from more intuitive explanations or additional context to help non-specialist readers engage more effectively with the material.
Response: Thank you for your feedback. We have included an extra definition of the Elotope as well as a diagram. Hopefully this might help the non-specialist

understand its meaning.

Despite these issues, the manuscript offers a meaningful and rigorous contribution to the study of rating systems. The results are valuable and practically relevant.

Response: Thank you for acknowledging the relevance of our work.

I recommend a careful round of language and formatting revisions. Once these are addressed, the manuscript will be well positioned for publication.

Response: Thank you for your recommendation. We have carefully read through the manuscript and corrected any language-based errors.

4 Reviewer #3: Review Report for PONE-D-25-15647 "The Impact of Intransitivity on the Elo Rating System"

Overall Assessment

This paper makes valuable theoretical contributions to understanding Elo rating systems under intransitive conditions, particularly by providing new insights into the existence and uniqueness of equilibria, established through the application of Brouwer's theorem. Additionally, the analysis of the dependence on match-selection matrices marks a significant advance in the field. The introduction of the intransitivity metric, I(A), offers a promising new approach for evaluating ranking systems under conditions where traditional transitivity assumptions no longer hold. The contributions of this paper are especially important for ensuring fairness in competition, as they provide a more nuanced understanding of how rankings can be influenced by non-transitive relationships between competitors. By addressing the complexities that arise from intransitivity, the proposed framework allows for a more accurate and equitable representation of player abilities, ultimately fostering greater fairness in competitive environments. Below are suggestions aimed at strengthening clarity, empirical validation, and the broader impact of these findings.

4.1 Major Comments

1.Clarification of Seemingly Contradictory Contributions The paper presents two main results:

(i) Ratings depend on match selection (Q) under intransitivity (Section 4),

(ii) A unique equilibrium exists for any fixed Q (Sections 5–6).

At first glance, these findings may seem contradictory.

Suggestion: To address this, explicitly reconcile the two results in the abstract/introduction (e.g., "Although the final ratings are highly dependent on the match-selection distribution Q, we demonstrate that for any fixed Q, a unique equilibrium exists").

Response: Thank you for bringing this to our attention. We include the paragraph "To clarify, for every set of probabilities Q with which players are selected to play one another, there is a corresponding fixed point in the Elo rating system. The Elo ratings will home in on this point so long as pairs of players are being selected using Q. If Q were to change, then so too would the location of this fixed point". We have also clarified this point in the paper's abstract.
2.Theoretical Proofs: Opportunities for Refinement Theorem 5.3: The condition requiring the hypersphere radius (R) to be "sufficiently large" lacks precise quantitative bounds. Suggestion: Clarify how (R) depends on (η), (Q), or (P) (e.g., using Lemma 5.4) to enhance the rigor of the proof.
Response: The purpose of R is to demonstrate that the function f has a fixed point. It's precise value does not contribute to the proof. However, to address any lingering concerns we have included an algorithm to calculate a rough upper bound on R in the appendices.
Theorem 6.1: The proof of injectivity assumes ($Q_{ij} = 1$) for simplicity, but it overlooks more general cases of (Q) (p. 21). A more thorough discussion is needed regarding how the curvature of ($\sigma(\cdot)$) impacts injectivity. Suggestion: Include a remark on how nonlinearity affects orthant preservation, or reference differential geometry tools (e.g., transversality) for a deeper analysis.
Response: Our proof does consider the general cases where $Q_{ij} \neq 1$. This is included towards the end of the proof. We have added sentences to more clearly signpost this part of the proof. As for differential geometric tools, our proof only makes use of the idea of a tangent plane. The concept of transversality is not relevant to this proof, which we have attempted to make more clear.
3.Empirical Validation Gap The experiments primarily validate (I(A)) (Section 7), but they fail to demonstrate the fundamental phenomenon: match-dependent ratings under intransitivity (Section 4). Recommendation: Include a straightforward case study (e.g., 3-player cyclic wins) that illustrates how different (Q) (e.g., chain vs. star match patterns) result in varying Elo ratings. This would provide empirical support for Theorem 4.1.
Thank you for this comment. Response: We have included a straightforward case study in the end of Section 7. This demonstrates the Elotope as well as the dependence of Q for the final Elo ratings.

4.2 Minor Comments

1.Introduction: This section introduces the Bradley-Terry model and its connection to the Elo rating system, explaining how the Elo system is applied in various competitive contexts. While the background and history of the Elo system are clearly presented, the explanation could place more emphasis on why the study specifically focuses on intransitivity phenomena.
Response: We have included the following paragraph in the Introduction section. "We focus on the phenomenon of intransitivity because it has largely been neglected in previous research on the Elo rating system. The authors have not been able to find any mathematically rigorous analysis of how the Elo rating

system behaves on data that displays intransitivity and are writing this paper to bridge this existing gap in the literature".
It would also be helpful to highlight the practical implications for fields like eSports, sports events, and machine learning. While the theoretical framework is well outlined, providing a more intuitive explanation of intransitivity and briefly mentioning real-world applications (such as in competitive games or sports rankings) would strengthen the argument.

Overall, the introduction sets up the research well, but simplifying the language and making clearer connections to practical uses would enhance its persuasiveness.
Response: the importance of theoretical research like this is to place Elo rating system on firm mathematical foundations. This understanding of how Elo ratings behave is what gives us the ability to trust our conclusions drawn from the algorithm. This is the practical application of the paper. We have altered the introduction section to make this point more clearly.
2.Terminology and Notation The term "Elotope" (p. 15) is introduced without a formal definition. Suggestion: Provide a definition early on, for example, "the set of all possible final ratings under varying Q."
Response: We have included the definition: The Elotope can be defined as the image of the function that sends a selection matrix Q to its corresponding final Elo score. and have signposted this definition earlier in the paper. We have also included a diagram of an Elotope to help make the exposition more understandable.
There is inconsistent notation, such as grad(r) vs. ∇r (e.g., Eq 4–5), and $\sigma(\cdot)$ vs. $logit(\cdot)$. Suggestion: Standardize the notation to ∇r and $\sigma(\cdot)$ throughout the paper.
Response: There are no longer any nablas in the paper. We make the connection between logit and sigma once and use sigmas from then on.
3.Figures and Accessibility Figures 6–9: Axes are unlabeled (e.g., "Number of Samples" in Fig 6).
Response: We have labelled the axes of our figures.
Figure 5: Simplex vertices should label strategies (R/P/S).
Response: The simplex vertices have been labelled R, P, and S in Figure 5.
Suggestion: Label axes clearly in all figures and add strategy labels to Fig 5.
Response: We have made these changes. Thankyou
Strengths Theoretical Rigor: The application of Brouwer's theorem and Hodge decomposition is elegant. Novel Metric: I(A) cleverly utilizes cyclic/transitive components. Impactful Rebuttal: The paper effectively addresses Balduzzi et al. [ ] by showing partial Elo robustness (p. 11). Suggested Revisions Summary Clarify the relationship between match-dependence and equilibrium uniqueness. Tighten the hyperradius bounds in Theorem 5.3 and discuss the impact of $\sigma(\cdot)$ in Theorem 6.1. Provide an empirical demonstration of Q-dependent ratings. Standardize notation and define "Elotope" upon first use. Improve figure labels and share the simulation code. This work significantly contributes to rating system theory, and with minor adjustments, its impact will be even greater. I commend the authors for their rigorous analysis and look forward to their re-

sponse to these suggestions.
Response: We have addressed all of the suggested revisions in the list.
