# Supplementary material for: The impact of intransitivity on the Elo rating system
Source: PLoS One. 2025 Dec 26;20(12):e0338261. doi: 10.1371/journal.pone.0338261 (PMC12742789; doi:10.1371/journal.pone.0338261)
Supplement: Attachment — Submitted filename: Review Report for PONE-D-25-15647.docx [file pone.0338261.s002.docx]

**Review Report for PONE-D-25-15647**
"The Impact of Intransitivity on the Elo Rating System"

### ****Overall Assessment****

This paper makes valuable theoretical contributions to understanding Elo rating systems under intransitive conditions, particularly by providing new insights into the existence and uniqueness of equilibria, established through the application of Brouwer’s theorem. Additionally, the analysis of the dependence on match-selection matrices marks a significant advance in the field. The introduction of the intransitivity metric, I(A), offers a promising new approach for evaluating ranking systems under conditions where traditional transitivity assumptions no longer hold.

The contributions of this paper are especially important for ensuring fairness in competition, as they provide a more nuanced understanding of how rankings can be influenced by non-transitive relationships between competitors. By addressing the complexities that arise from intransitivity, the proposed framework allows for a more accurate and equitable representation of player abilities, ultimately fostering greater fairness in competitive environments.

Below are suggestions aimed at strengthening clarity, empirical validation, and the broader impact of these findings.

### ****Major Comments****

1.Clarification of Seemingly Contradictory Contributions

The paper presents two main results:

(i) Ratings depend on match selection (Q) under intransitivity (Section 4),

(ii) A unique equilibrium exists for any fixed Q (Sections 5–6).

At first glance, these findings may seem contradictory.

Suggestion:To address this, explicitly reconcile the two results in the abstract/introduction (e.g., "Although the final ratings are highly dependent on the match-selection distribution Q, we demonstrate that for any fixed Q, a unique equilibrium exists").

2.Theoretical Proofs: Opportunities for Refinement

Theorem 5.3: The condition requiring the hypersphere radius ( R ) to be "sufficiently large" lacks precise quantitative bounds.

Suggestion: Clarify how ( R ) depends on ( \eta ), ( Q ), or ( P ) (e.g., using Lemma 5.4) to enhance the rigor of the proof.

Theorem 6.1: The proof of injectivity assumes ( Q_{ij} = 1 ) for simplicity, but it overlooks more general cases of ( Q ) (p. 21). A more thorough discussion is needed regarding how the curvature of ( \sigma(\cdot) ) impacts injectivity.

Suggestion: Include a remark on how nonlinearity affects orthant preservation, or reference differential geometry tools (e.g., transversality) for a deeper analysis.

3.Empirical Validation Gap

The experiments primarily validate ( I(A) ) (Section 7), but they fail to demonstrate the fundamental phenomenon: match-dependent ratings under intransitivity (Section 4).

Recommendation: Include a straightforward case study (e.g., 3-player cyclic wins) that illustrates how different ( Q ) (e.g., chain vs. star match patterns) result in varying Elo ratings. This would provide empirical support for Theorem 4.1.

### ****Minor Comments****

1.Introduction: This section introduces the Bradley-Terry model and its connection to the Elo rating system, explaining how the Elo system is applied in various competitive contexts. While the background and history of the Elo system are clearly presented, the explanation could place more emphasis on why the study specifically focuses on intransitivity phenomena. It would also be helpful to highlight the practical implications for fields like eSports, sports events, and machine learning. While the theoretical framework is well outlined, providing a more intuitive explanation of intransitivity and briefly mentioning real-world applications (such as in competitive games or sports rankings) would strengthen the argument. Overall, the introduction sets up the research well, but simplifying the language and making clearer connections to practical uses would enhance its persuasiveness.

2.Terminology and Notation

The term "Elotope" (p. 15) is introduced without a formal definition.

Suggestion: Provide a definition early on, for example, “the set of all possible final ratings under varying Q.”

There is inconsistent notation, such as grad(r) vs. ∇r (e.g., Eq 4–5), and σ(⋅) vs. logit(⋅).

Suggestion: Standardize the notation to ∇r and σ(⋅) throughout the paper.

3.Figures and Accessibility

Figures 6–9: Axes are unlabeled (e.g., "Number of Samples" in Fig 6).

Figure 5: Simplex vertices should label strategies (R/P/S).

Suggestion: Label axes clearly in all figures and add strategy labels to Fig 5.

### ****Strengths****

Theoretical Rigor: The application of Brouwer’s theorem and Hodge decomposition is elegant.

Novel Metric: I(A) cleverly utilizes cyclic/transitive components.

Impactful Rebuttal: The paper effectively addresses Balduzzi et al. [9] by showing partial Elo robustness (p. 11).

### ****Suggested Revisions Summary****

Clarify the relationship between match-dependence and equilibrium uniqueness.

Tighten the hyperradius bounds in Theorem 5.3 and discuss the impact of σ(⋅) in Theorem 6.1.

Provide an empirical demonstration of Q-dependent ratings.

Standardize notation and define "Elotope" upon first use.

Improve figure labels and share the simulation code.

This work significantly contributes to rating system theory, and with minor adjustments, its impact will be even greater. I commend the authors for their rigorous analysis and look forward to their response to these suggestions.
