# Supplementary material for: The impact of intransitivity on the Elo rating system
Source: PLoS One. 2025 Dec 26;20(12):e0338261. doi: 10.1371/journal.pone.0338261 (PMC12742789; doi:10.1371/journal.pone.0338261)
Supplement: Attachment — Submitted filename: Reviewer Comments.docx [file pone.0338261.s001.docx]

**Reviewer Comments**

The manuscript titled *"Balancing Food-Environment-Energy-Economic: A Multi-Criteria Assessment of Water and Fertilizer Strategies in Semi-Arid Sandy Loam Soil Agroecosystems"* presents a scientifically robust and methodologically sound evaluation of five water and fertilizer strategies. The study employs a comprehensive multi-criteria framework integrating Life Cycle Assessment (LCA), Emergy Analysis (EMA), and a Sustainability Performance Index (SPI), addressing key dimensions of sustainable agriculture: food production, environmental protection, energy use, and economic return. The experimental design is well-structured, and the results are clearly presented and statistically validated.

The findings are highly relevant to the *Journal of Cleaner Production*, especially given the urgent need for efficient resource management in dryland agroecosystems. The authors' integration of SPI for sustainability trade-off analysis across treatments is novel and valuable.

However, several **areas could be improved** to enhance clarity and completeness:

- **Abstract**: Overly dense and slightly repetitive; simplify and reduce jargon for broader readability.
- **Discussion section**: Some overlap with the results; clearer distinction between result interpretation and broader implications is needed.
- **Language and style**: Some long and complex sentences throughout the manuscript—especially in the introduction and discussion—should be revised for better clarity and flow.
- **Justification of weights in SPI**: The choice of equal weighting for the four dimensions is reasonable but would benefit from a brief explanation or reference; consider noting this limitation.
- **Policy relevance**: While government subsidies and incentives are discussed, a short reflection on the broader applicability of results without policy support would strengthen the practical implications.

In summary, the manuscript is of high quality and contributes meaningfully to the literature on sustainable agricultural intensification. I recommend **acceptance with minor revisions**, focusing on improving clarity, tightening the narrative, and addressing the points listed above.
